# Brain oxygen responses induced by opioids: focus on heroin, fentanyl, and their adulterants

**DOI:** 10.3389/fpsyt.2024.1354722

**Published:** 2024-01-17

**Authors:** Eugene A. Kiyatkin, Shinbe Choi

**Affiliations:** Behavioral Neuroscience Branch, National Institute on Drug Abuse–Intramural Research Program, National Institutes of Health, DHHS, Baltimore, MD, United States

**Keywords:** opioids, health complications, brain hypoxia, metabolic brain activation, peripheral vasoconstriction, cerebral vasodilation, nucleus accumbens, rats

## Abstract

Opioids are important tools for pain management, but abuse can result in serious health complications. Of these complications, respiratory depression that leads to brain hypoxia is the most dangerous, resulting in coma and death. Although all opioids at large doses induce brain hypoxia, danger is magnified with synthetic opioids such as fentanyl and structurally similar analogs. These drugs are highly potent, act rapidly, and are often not effectively treated by naloxone, the standard of care for opioid-induced respiratory depression. The goal of this review paper is to present and discuss brain oxygen responses induced by opioids, focusing on heroin and fentanyl. In contrast to studying drug-induced changes in respiratory activity, we used chronically implanted oxygen sensors coupled with high-speed amperometry to directly evaluate physiological and drug-induced fluctuations in brain oxygen levels in awake, freely moving rats. First, we provide an overview of brain oxygen responses to physiological stimuli and discuss the mechanisms regulating oxygen entry into brain tissue. Next, we present data on brain oxygen responses induced by heroin and fentanyl and review underlying mechanisms. These data allowed us to compare the effects of these drugs on brain oxygen in terms of their potency, time-dependent response pattern, and potentially lethal effect at high doses. Then, we present the interactive effects of opioids during polysubstance use (alcohol, ketamine, xylazine) on brain oxygenation. Finally, we consider factors that affect the therapeutic potential of naloxone, focusing on dosage, timing of drug delivery, and contamination of opioids by other neuroactive drugs. The latter issue is considered chiefly with respect to xylazine, which strongly potentiates the hypoxic effects of heroin and fentanyl. Although this work was done in rats, the data are human relevant and will aid in addressing the alarming rise in lethality associated with opioid misuse.

## 1 Introduction

Opioids are a dangerous class of addictive drugs with high incidence rates of serious health complications resulting from drug overdose (1–6). In addition to heroin, which has been circulating illicit drug markets for decades, fentanyl and structurally similar synthetic opioids have increasingly become an area of concern for both the public and medical community due to abnormally high overdose-induced lethality (7, 8). According to the most recent statistics, the number of opioid-involved overdose deaths in the United States has rapidly increased from 21,089 in 2010 to 110,000 in 2022 (9, 10), highlighting a major need for better strategies to treat and care for individuals facing opioid-related health complications.

The opioid crisis is compounded by the issue of polysubstance use. Due to financial or social barriers, heroin or fentanyl are rarely accessible at pure or pharmaceutical grade. As such, they are often contaminated or used with other neuroactive drugs that increase the potency of the primary drug. Analyses of drug samples from the drug market and intoxicated patients have revealed several commonly co-administered drugs, including alcohol, ketamine, benzodiazepines, and xylazine (11, 12). Hence, we focus on exploring the interactions between opioids and these other substances.

In this work, we present and discuss our recent data exploring physiological and drug-induced changes in brain oxygen levels obtained by using oxygen sensors coupled with high-speed amperometry in freely moving rats. We focused on oxygen monitoring because opioids are known to induce respiratory depression and resulting brain hypoxia, which is thought to be the primary cause of death from opioid overdose (13–16). Furthermore, brain oxygen is an important homeostatic parameter, and its fluctuations have profound effects on metabolic activity and proper functioning of brain cells. In contrast to plethysmography and pulse oximetry, which are often used to quantify drug-induced respiratory depression (13, 17–21), our electrochemical technology allows direct evaluation of oxygen fluctuations in the brain’s extracellular space that are induced by natural sensory stimuli or drugs under physiologically relevant conditions at a second-scale temporal resolution. Although we primarily focused on brain oxygen, simultaneous oxygen monitoring in the brain and subcutaneous (SC) space allowed us to examine the relationship between oxygen fluctuations in the brain and periphery, elucidating the mechanisms underlying brain oxygen responses.

Opioids like fentanyl and heroin interact with the same receptor targets stimulated by endogenous opioid peptides, sharing the underlying mechanisms of physiological brain oxygen responses. Therefore, first, we discuss our data on physiological fluctuations in brain oxygenation induced by various sensory stimuli and during motivated behavior. Here, we also present available data on the mechanisms mediating brain oxygen responses, specifically the role of local and generalized neural activation and central or peripheral vascular changes. Second, we present the data on brain oxygen responses induced by heroin and fentanyl, discussing both similarities and differences. Through our electrochemical technique, we revealed differences between the opioid-induced brain oxygen responses and the pattern of respiratory depression as assessed by monitoring respiratory activity. Furthermore, we report that both opioids at low doses may induce opposing responses in the brain and peripheral tissues, thus clarifying important role of peripheral vasoconstriction in mediating brain oxygen responses.

Third, to address poly-drug use, we discuss the findings of our recent studies on the interaction of heroin and fentanyl with other neuroactive substances, including alcohol, ketamine, and xylazine. We examined the effects of these drugs on brain oxygen when they are used both alone and co-administered with heroin or fentanyl. Fourth, although naloxone is the best tool for reversing the hypoxic effects of opioids, clinical data indicate that it is less effective in attenuating the hypoxic effects of fentanyl and other fentanyl-like synthetic drugs (22, 23). Hence, to understand the cause of this diminished efficacy, we discuss our recent data that suggests that the therapeutic potential of naloxone is critically dependent on the time interval between the onset of opioid overdose symptoms and initiation of naloxone treatment. Fifth and finally, we discuss the treatment potential of naloxone when heroin or fentanyl is contaminated by or mixed with xylazine, a non-opioid veterinary drug that is increasingly being found as an adulterant in opioid supplies across the United States (24, 25). Although our data were obtained in rats, they are clinically relevant, showing both the mechanisms underlying the adverse effects of opioids and proposing possible treatment strategies to block or minimize these adverse effects.

## 2 Physiological fluctuations in brain oxygenation and their mechanisms

The metabolic activity of brain cells critically depends on the proper delivery of oxygen, which arrives at brain tissue from arterial blood, where its concentration is higher than in the brain’s extracellular space. Therefore, brain oxygen levels directly depend on blood supply, which is tightly regulated and remains relatively stable in healthy organisms under physiologically relevant conditions. Oxygenation of arterial blood is maintained by respiration (breathing), depending on oxygen content in the inspired air and the efficiency of respiratory activity, which determines the oxygen transfer from the external medium to the lung’s vessels. Respiration is a sensitive physiological parameter, with its efficiency increasing during physiological and behavioral activation (26), while decreasing following exposure to sedative drugs including opioids (13, 14, 16). While respiration-dependent changes in blood oxygen content modulate oxygen entry into brain tissue, other factors like simultaneous changes in neuronal activity, brain metabolism, and the tone of cerebral vessels also provide significant contributions to changes in brain oxygenation.

Although gradient-dependent entry of oxygen serves as the primary mechanism determining oxygen levels in the brain’s extracellular space, numerous data suggest that oxygen entry is modulated by neural activity via changes in the cerebral vessel tone and fluctuations in cerebral blood flow (27–30). This gradient-independent neural mechanism, usually considered in terms of neurovascular coupling, allows the brain to receive more oxygen in advance of its enhanced use during functional neural activation, preventing any possible metabolic deficit that may harm brain cells. The enhanced inflow of oxygen into the brain’s extracellular space is opposed by continuous consumption, tending to decrease brain oxygen levels. Oxygen consumption is also increased during functional neural activation (31–33), which also affects the levels of this substance in brain tissue. While it is well established that the extracellular levels of oxygen reflect a balance of highly dynamic and opposing influences, understanding of the physiological fluctuations of this important homeostatic parameter remains limited.

**Figure 1:**
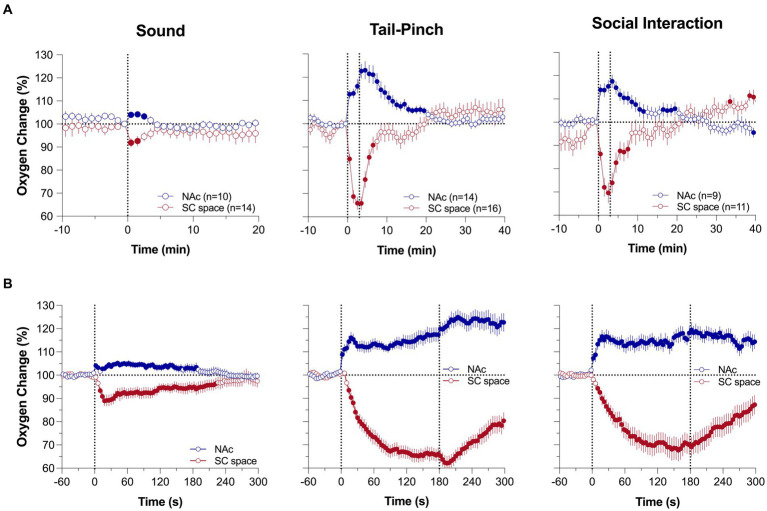
Mean (±SEM) changes in oxygen levels in the NAc and SC space induced by 1-s audio stimulus, 3-min tail-pinch, and 3-min social interaction. Data are shown as percent change vs. pre-stimulus baseline (=100%). **(A)** shows data analyzed with 1-min time-resolution and **(B)** shows the same data analyzed with 10-s time resolution. n is the number of averaged responses. [Modified from Thomas et al. (37)].

To determine how brain oxygenation fluctuates under physiological conditions, we examined the changes in oxygen levels in the nucleus accumbens (NAc) [a critical structure in sensory motor integration (34, 35)] and subcutaneous (SC space; a densely vascularized area with no metabolic activity of its own) induced by various natural arousing stimuli (36, 37). Consistent with early findings using electrochemical oxygen sensors (38, 39), we found that NAc oxygen levels rapidly increase following exposure to various arousing stimuli (Figure 1A). These increases are relatively small in magnitude (1–4 μM or ~ 5–20% over the baseline), with the most rapid acceleration immediately after the stimulus onset. These increases are exceptionally rapid, becoming significant within the first 10 s following the stimulus onset (Figure 1B). Brain oxygen responses also differed depending upon the stimulus duration and its presumed biological significance. A short auditory stimulus induced the weakest change, which was evident within ~2–3 min, but both the 3-min tail-pinch and 3-min social interaction induced stronger and more prolonged oxygen increases that peaked at the end of or after the offset of stimulus exposure. These phasic responses elicited by arousing stimuli were superimposed over spontaneous fluctuations in basal oxygen levels, which were often equally rapid and strong as those induced by arousing stimuli. In contrast to the brain, SC oxygen levels rapidly and strongly decreased, negatively correlating with increases in brain oxygen levels (Figure 1). These changes were also rapid and larger in amplitude and duration than in the brain. Therefore, we found that modest increases in brain oxygen levels (functional hyperoxia) co-exist with stronger oxygen decreases in peripheral tissues (functional peripheral or systemic hypoxia).

**Figure 2:**
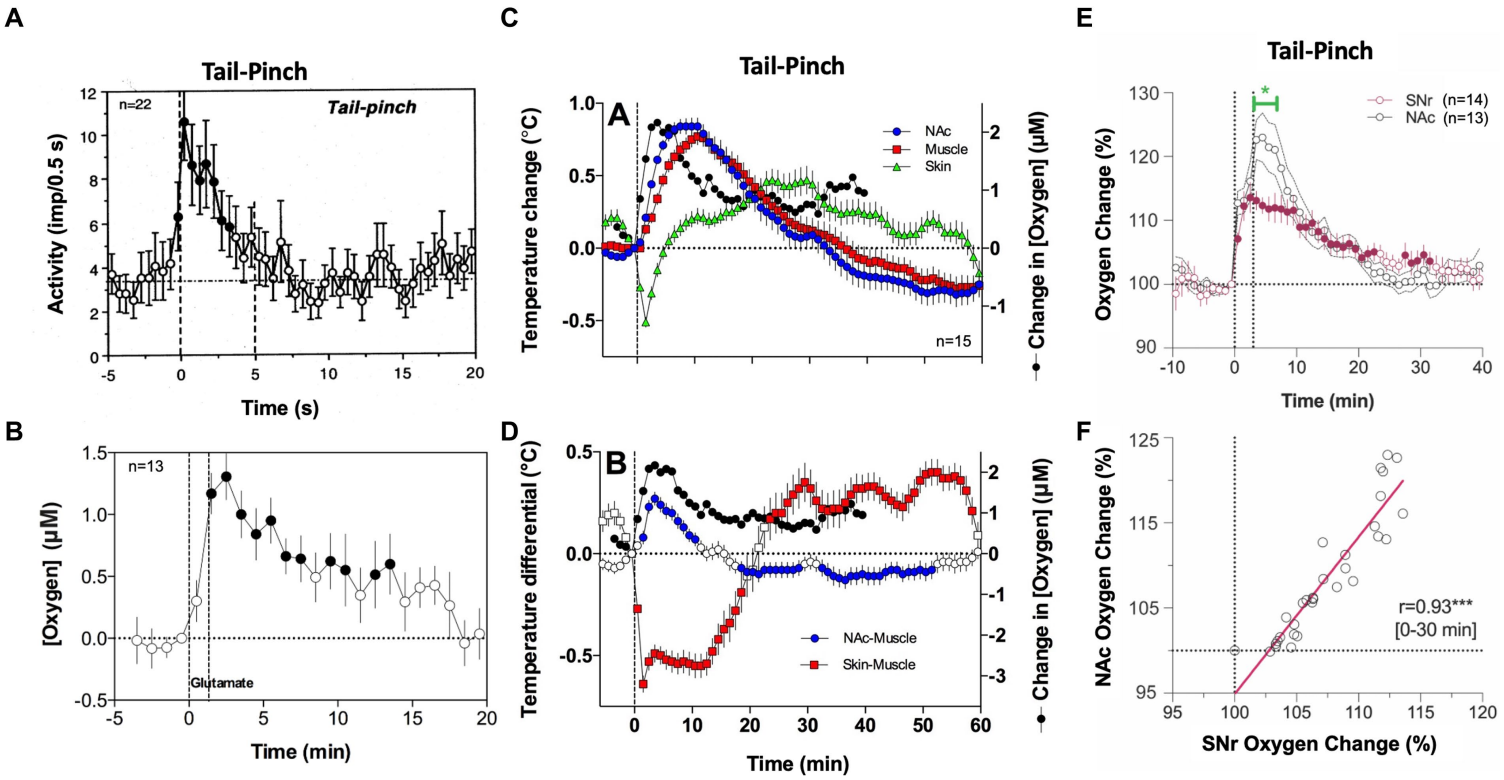
The role of neural activation and vascular response in triggering physiological increases in brain oxygenation. **(A)** Mean changes in impulse activity of accumbal neurons induced by 5-s tail-pinch. **(B)** Changes in NAc oxygen levels induced by local microinjection of glutamate nearby the recording side. **(C)** Changes in temperature in the NAc, temporal muscle, and SC space induced by 3-min tail-pinch shown together with changes in NAc oxygen levels. **(D)** Changes in NAc-muscle and skin-muscle temperature differentials induced by tail-pinch shown together with changes in oxygen levels. **(E)** Changes in oxygen levels induced by 3-min tail-pinch in the NAc and SNr. Green line shows between-structure differences. **(F)** Correlative relationships between brain oxygen changes induced by tail-pinch in the NAc and SNr, r = coefficient of correlation. Despite differences in response magnitude, changes in both structures were highly correlative (*r* = 0.93). Original data were presented in Solis et al. (36), Thomas et al. (37), Solis et al. (44) and Kiyatkin and Brown (45).

The rapidity of brain oxygen increases points to neuronal activation as the triggering force. This mechanism is consistent with electrophysiological data obtained in dorsal and ventral striatal neurons in awake, freely moving rats. These cells have low, sporadic activity at rest and are phasically excited by various arousing stimuli (40–43). As shown in Figure 2A, a short, 5-s tail-pinch induced phasic increases in NAc neuronal spiking activity that peaked within the first seconds following the stimulus onset, suggesting its role in triggering subsequent increases in brain oxygenation. The neuronal triggering mechanism was confirmed by recording brain oxygen responses induced by local microinjection of glutamate near the oxygen sensing area [(36), Figure 2B]. Accumbal neurons are universally sensitive to both locally applied or iontophoretically delivered glutamate that result in their phasic excitations (41, 42). When injected at the optimal concentration (2–5 μM), glutamate microinjections increase NAc levels of oxygen. In contrast to responses elicited by natural arousing stimuli, glutamate-induced oxygen increases exhibited longer and more variable onset latencies with relatively small magnitudes, which were similar to or lower than those seen with the natural arousing stimuli. Therefore, local activation of accumbal neurons induced by arousing stimuli contributes to local cerebral vasodilation that enhances oxygen entry into the brain’s extracellular space.

However, simultaneous oxygen recordings from the NAc and SC space revealed another contributor to the brain’s hyperoxic responses. Since oxygen levels in arterial blood remain stable or slightly increase following sensory stimulation, a strong decrease in oxygen in the SC space can occur only due to skin vasoconstriction. This mechanism was confirmed by our thermorecording studies that revealed similar dissociative temperature responses induced by arousing stimuli: increases in the NAc and decreases in the SC space (46). As shown in Figure 2C, temperature increases induced by tail-pinch mirror brain oxygen increases but occur with longer onset latencies and are more prolonged than changes in oxygen. An even stronger correlation was found between changes in NAc oxygen and NAc-muscle temperature differentials, a measure of metabolic brain activation (Figure 2D). Tail-pinch also induced a strong decrease in the skin-muscle differential, indicating skin vasoconstriction. In this case, the oxygen decrease was more rapid than the changes in brain temperature, again suggesting neuronal triggering.

The increases in brain temperature and oxygenation induced by salient sensory stimuli are different manifestations of generalized arousal, an organism’s adaptive response to salient environmental challenges. While generalized and structure-specific changes in neuronal activity contribute to increased brain oxygenation, skin vasoconstriction appears to be another contributor. This vasoconstriction is a centrally mediated effect and a component of sympathetic activation. By constricting peripheral blood vessels, blood is re-distributed from the peripheral domain to the central domain, with subsequent cerebral vasodilation and increased cerebral blood flow. Decreasing blood flow in the skin allows more arterial blood to enter brain tissue (functional hyperemia), supplying more oxygen and glucose necessary for enhanced metabolic activity. Thus, it appears that peripheral vasoconstriction has a dual function: inducing brain hyperthermia by inhibiting heat dissipation, and enhancing supply of oxygen and glucose to the brain.

In contrast to the direct effects of structure-specific neuronal activation, the vascular effect appears to be more global, primarily dependent on the density of vascularization in brain tissue. As most gray matter is uniform in vascular density (47), we predicted that increases in oxygenation will be similar across different brain structures despite different changes in neuronal activity. To explore this hypothesis, we compared oxygen changes induced by tail-pinch in the NAc and *substantia nigra, pars reticulata (SNr)*, two distantly located brain structures that have profound differences in spontaneous and stimulus-induced impulse activity (38). SNr neurons have high impulse activity and are inhibited by sensory stimuli (48–50), but NAc neurons have low, sporadic activity and are excited by sensory stimuli (40–42). Furthermore, NAc neurons are highly sensitive to glutamate, but most SNr cells are insensitive to glutamate and instead highly sensitive to GABA, which inhibits their impulse activity (50). Despite these differences in neuronal activity, the pattern of oxygen response was similar in both structures (Figure 2E), and oxygen changes were highly correlative (Figure 2F).

## 3 Brain oxygen responses induced by iv heroin and fentanyl: common features, differences, and underlying mechanisms

Mu-opioid receptors, densely expressed in the brain, spinal cord, and peripheral tissues, are the primary target of opioid drugs, including heroin, fentanyl, and related derivatives. Selective and excessive stimulation of opioid receptors by these opioid drugs results in abnormal physiological and behavioral effects. Among these effects, respiratory depression, with subsequent robust decreases in the brain’s oxygen levels (brain hypoxia) is the most dangerous. However, by acting via the same receptor sites and involving similar mechanisms, changes in brain oxygen induced by opioid drugs at low doses should have certain similarity with physiological oxygen fluctuations elicited by natural arousing stimuli. Since opioid drugs induce excessive or abnormal stimulation of opioid receptors, drug-induced responses should also have essential differences from oxygen responses induced by natural stimuli.

**Figure 3:**
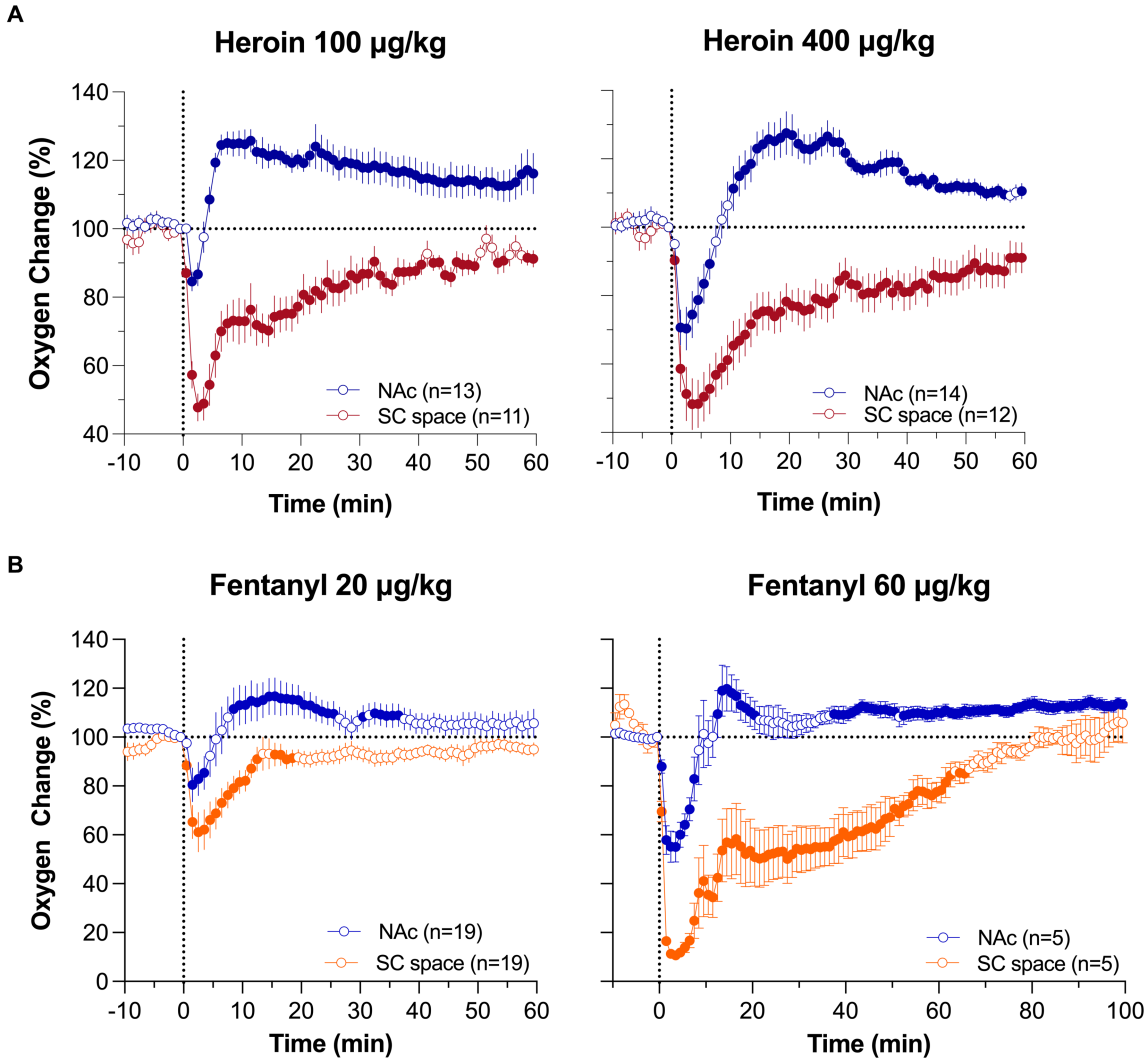
Mean (±SEM) changes in oxygen levels in the NAc and SC space induced by iv heroin **(A)** and fentanyl **(B)** at different doses. Filled symbols show values significantly different from pre-injection baseline. Original data were published in Thomas et al. (37) and Curay et al. (51).

Figure 3 shows mean changes in oxygen in the NAc and SC space induced by iv heroin and fentanyl. As can be seen in Figure 3A, heroin in both doses induces a biphasic oxygen change in the NAc, with a rapid and relatively transient decrease followed by a weaker and more prolonged increase. This immediate decrease was weak at a 0.1 mg/kg dose, which is optimal for heroin self-administration in rats (52) and within the range of human consumption (~7 mg/70 kg), but becomes stronger at a 0.4 mg/kg dose, which is near the upper limit of human consumption or overdose (~28 mg/70 kg). In contrast, oxygen levels in the SC space monophasically decreased, and these decreases were equally rapid, but stronger and more prolonged than in the brain.

Despite fentanyl’s higher potency, the pattern of oxygen response induced by iv fentanyl was similar to that induced by heroin (Figure 3B), with a biphasic change in the brain and monophasic decrease in the SC space. Drug-induced hypoxia was weak and transient at a lower dose (20 μg/kg or 1.4 mg/70 kg), but stronger and more prolonged at a higher dose (60 μg/kg or 4.2 mg/70 kg). Similar to heroin, oxygen decrease in the periphery was much stronger and more prolonged than in the brain, where it was evident for only ~5 and ~ 10 min at 20 and 60 μg/kg doses, respectively.

**Figure 4:**
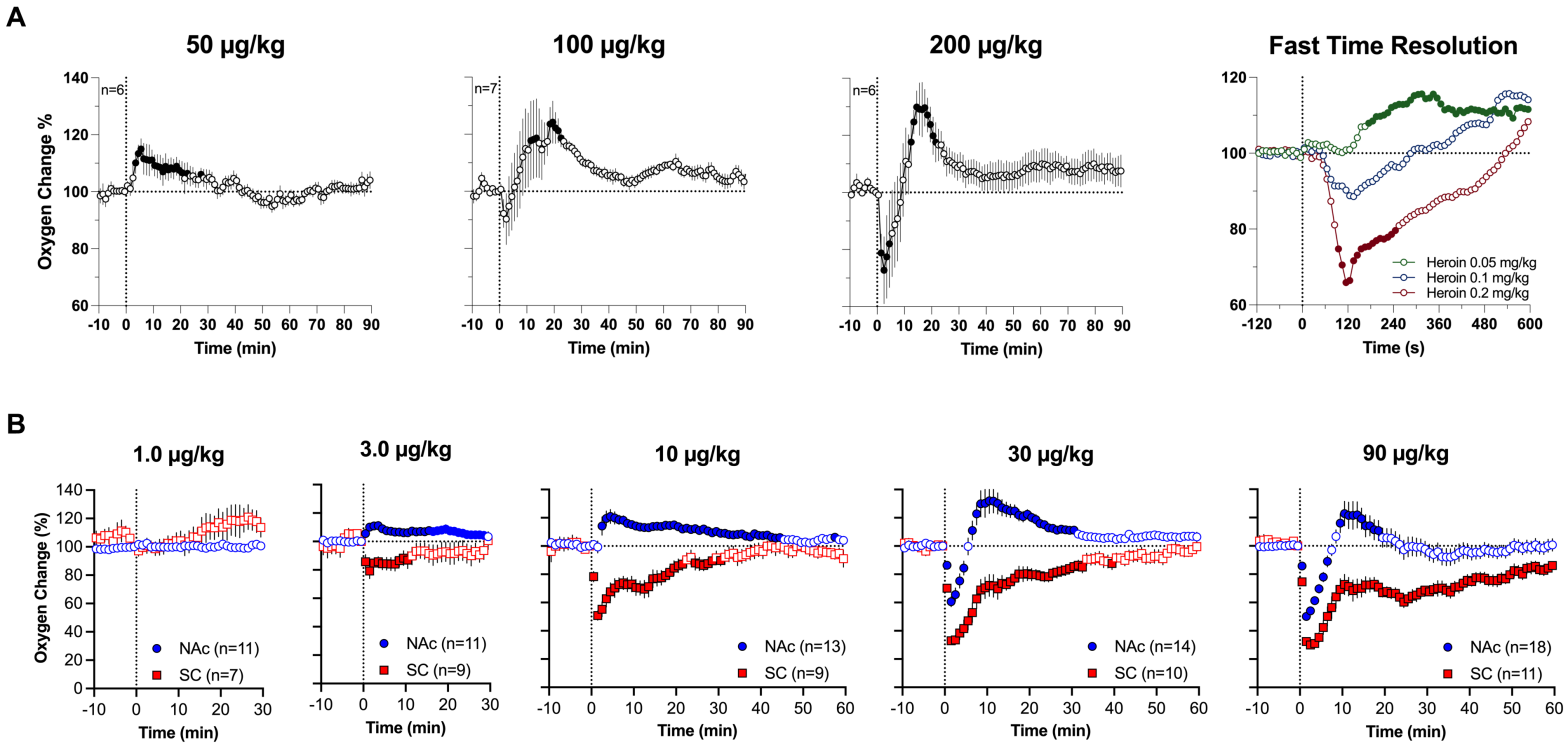
Mean (±SEM) changes in oxygen levels induced by iv heroin **(A)** and fentanyl **(B)** at different doses. **(A)** shows changes in the NAc, **(B)** shows changes in the NAc and SC space. n is the number of averaged responses. Original data were published in Thomas et al. (37).

Although respiratory depression is the primary cause of drug-induced brain hypoxia, the mechanisms underlying the post-hypoxic rebound-like hyperoxia, which occurs only in the brain and is absent in the periphery, are less clear. Since respiratory depression results in intra-cerebral accumulation of CO_2_, a powerful vasodilator (53–55), cerebral vasodilation and increased global cerebral blood flow may account for the post-hypoxic hyperoxia, as an adaptive mechanism opposing oxygen decreases induced by respiratory depression. Our dose–response tests allowed us to further explore this mechanism (Figure 4). We found that iv heroin at a lower, reinforcing dose (0.05 mg/kg) increased brain oxygen levels without any evidence of hypoxia (Figure 4A). When the dose of heroin was doubled (0.1 mg/kg), we observed a weak transient hypoxia followed by a larger oxygen increase. The hypoxic effect was stronger and more prolonged with further doubling of the dose (0.4 mg/kg). Profound differences in the initial effects of heroin were evident when data were analyzed with high temporal resolution. In this case, brain oxygen levels began to increase after heroin injection at a lowest dose with 150-200-s latency, but the oxygen decrease after the largest dose began with shorter, 50-60-s latencies. A similar pattern of dose–response changes was found with fentanyl. At low doses (3–10 ug/kg), injection of fentanyl only increased oxygen levels in the NAc, and decreased oxygen levels in the SC space (Figure 4B). Therefore, we found that oxygen responses to heroin and fentanyl injections manifest differently depending on drug dose.

**Figure 5:**
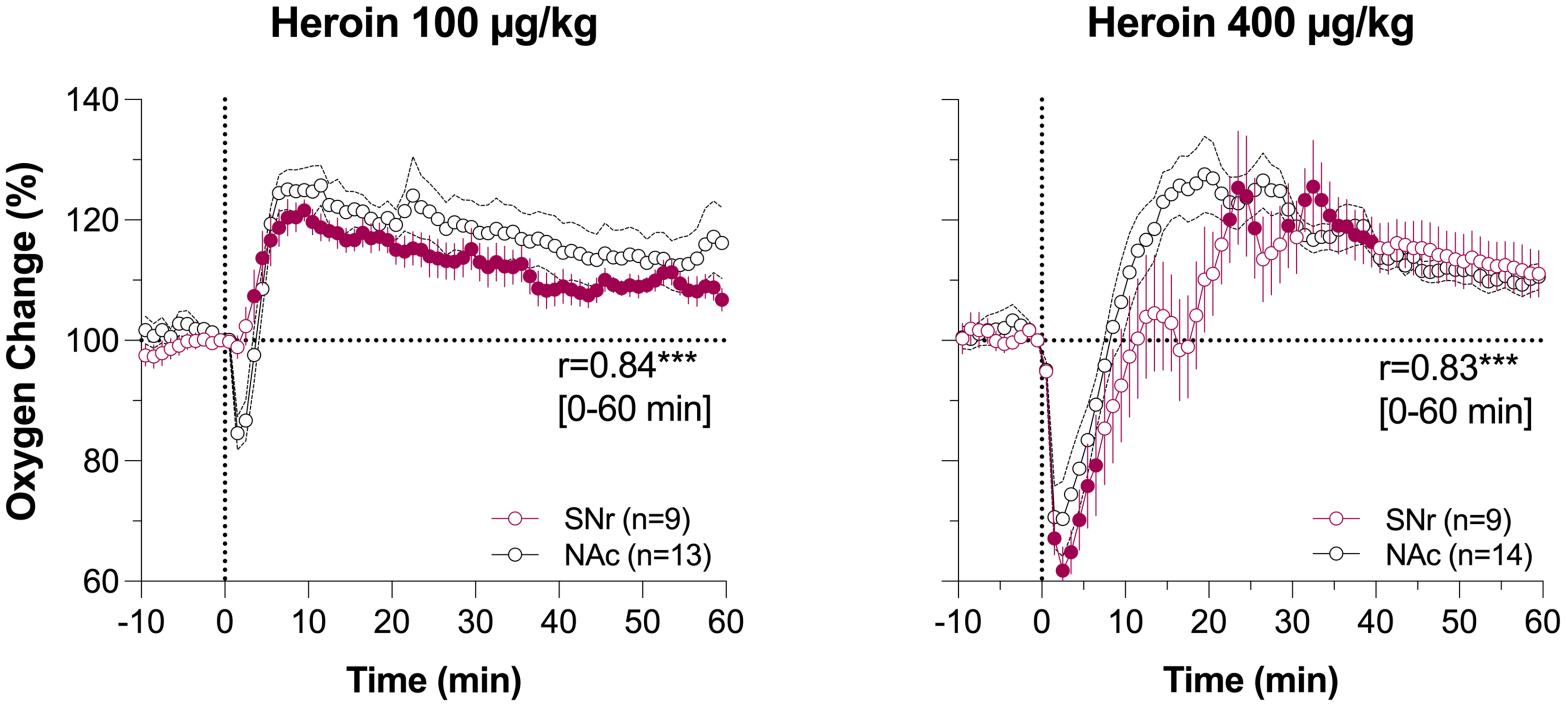
Mean changes in oxygen levels induced by iv heroin in the NAc (black) and SNr (red). Original data were published in Thomas et al. (37). Filled symbols for SNr show values significantly different from pre-injection baseline.

We further explored structure-specific oxygenation in response to drug stimuli by comparing oxygen responses induced by heroin at two doses in the NAc and SNr (37). As expected, due to the relatively similar nature of brain vascularization in deep brain structures, oxygen responses in these structures were very similar (Figure 5). A minimal between-structure difference was seen only at a low dose of heroin. There was no initial decrease in the SNr, while a decrease was evident in the NAc. Therefore, like physiological changes in brain oxygen, oxygen responses induced by opioids appear to be similar across distantly located brain structures containing neurons with different electrophysiological properties.

## 4 Interaction of heroin and fentanyl with other neuroactive substances

Heroin and fentanyl are rarely administered at pharmacological grade purity. Instead, they are often used or contaminated with other neuroactive substances that modify or amplify the effects of opioid drugs. To explore the complications that result from poly-drug use, we have conducted several experiments examining the effects of these neuroactive substances on brain oxygenation patterns induced by heroin and fentanyl.

### 4.1 Ethyl alcohol–heroin

Alcohol is the most commonly used psychoactive drug, which is often used with opioid drugs (56). Clinical reports reveal that patients admitted in emergency departments with a diagnosis of opioid overdose and symptoms of oxygen deficiency often have different amounts of alcohol in their blood (57). As alcohol induces CNS depression and modest respiratory depression at higher doses (58–60), it presents a risk of exacerbating the hypoxic effects of opioid drugs. To explore this, we examined the effects of alcohol at different doses on brain oxygenation and its effects on brain oxygen responses induced by iv heroin (61).

In contrast to other drugs, which can be delivered via subcutaneous, intraperitoneal or iv injection, alcohol is consumed orally. Hence, its physiological effects result from rapid diffusion into the blood stream from the stomach and duodenum, which are densely vascularized. To mimic this mechanism, we employed a chronically-implanted intra-gastric (ig) catheter that allowed for stress- and cue-free drug delivery. The doses of alcohol chosen in our study (0.5–2.0 g/kg or 35–140 g/70 kg) are equivalent to 87 and 350 mL of vodka (40% ethyl alcohol) and were delivered in a 20% solution. Next, heroin in these experiments was delivered at a 0.2 mg/kg iv dose, which is known to induce a well-defined biphasic oxygen response (see 2. above).

**Figure 6:**
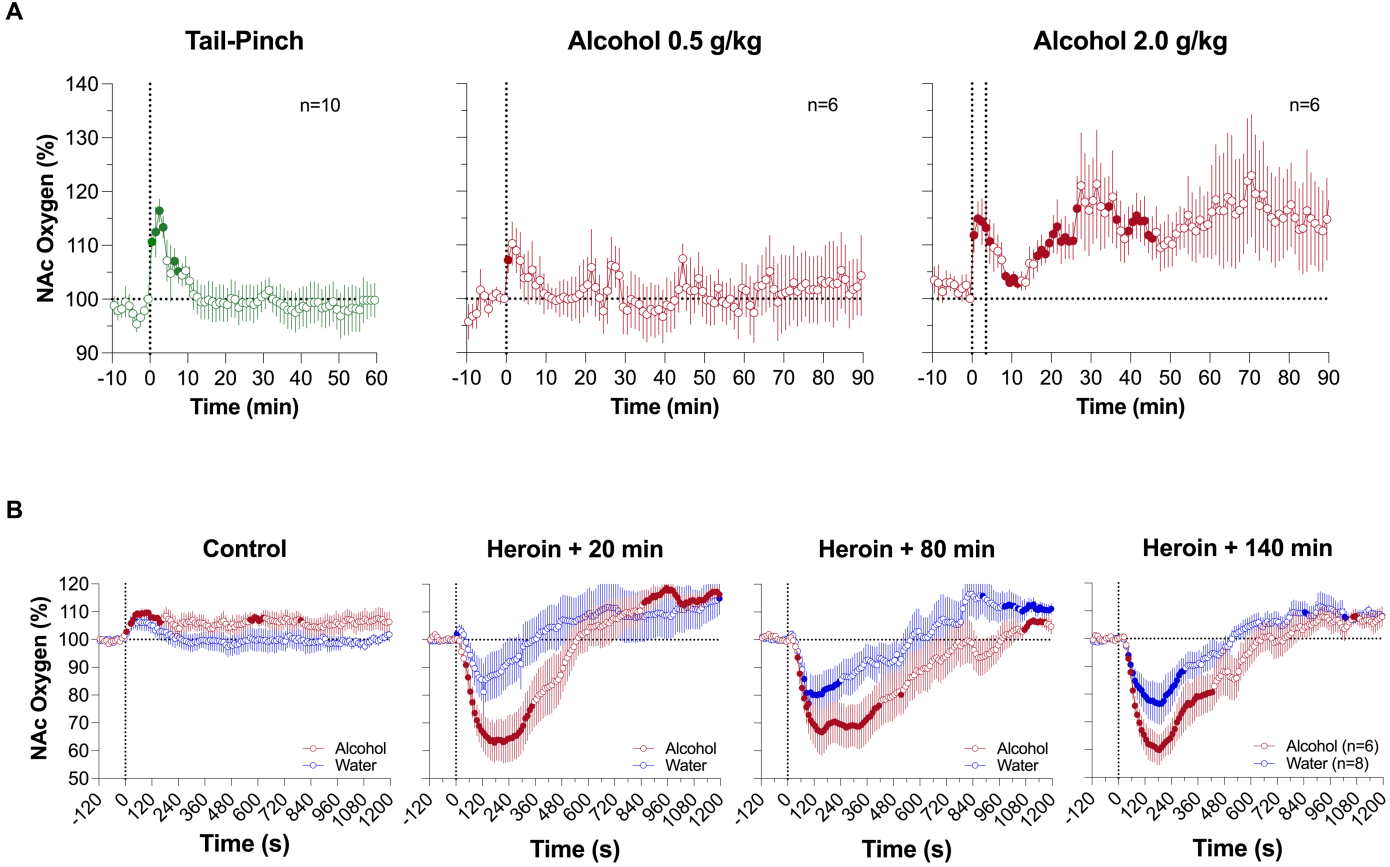
Mean changes in NAc oxygen levels induced by intragastric injection of ethyl alcohol at different doses **(A)**. **(B)** shows changes induced by iv saline and alcohol at 2.0 g/kg dose and changes induced by three heroin injections (0.2 mg/kg) delivered 20, 80 and 140 min after alcohol administration. Original data were published in Thomas et al. (61).

As shown in Figure 6A, ig alcohol delivery induced a rapid increase in NAc oxygenation. At a lower dose (0.5 g/kg), the effect was monophasic, but at higher dose (2 g/kg), the increase was biphasic, with a rapid and strong increase followed by a more prolonged, tonic increase. Since alcohol was delivered directly into stomach under stress-free conditions, short-latency phasic oxygen increases likely result from direct stimulation of the sensory fibers that densely innervate the stomach walls. This direct action of alcohol results in an afferent signal that ascends to the CNS via the celiac and superior cervical ganglions and the spinal cord, inducing neural activation. It is not known which types of receptors can be stimulated by alcohol but chemoreceptors and mechanoreceptors sensitive to pressure and stomach extension appear to be the likely candidates.

The second component of the NAc oxygen response, the tonic increase, was also an unexpected finding, and its mechanisms remain unclear. Interestingly, this change was associated with decreases in brain temperature and brain-muscle differentials (61), markers of central inhibition (62). We propose cerebral vasodilation and increased cerebral blood flow as a potential mechanism, since they are known effects of alcohol (63–65). Oxygen diffusion is gradient-dependent, and cerebral vasodilation followed by increased cerebral flow enhances oxygen delivery from arterial blood.

In comparing oxygen responses induced by heroin (0.2 mg/kg) after alcohol (2 g/kg) and water administration, oxygen decreases were stronger and more prolonged after ig alcohol administration (2 g/kg) (Figure 6B). The potentiating effect was strongest when heroin was injected 20 min after alcohol, and became weaker, but still significant, when heroin was delivered 80 and 140 min after alcohol. An area under the curve (AUC) analysis for oxygen decreases revealed that the initial brain hypoxia becomes 2 to 3-fold stronger in rats in the alcohol group than in rats in the water control group. Therefore, consistent with clinical observations (66, 67), alcohol potentiates heroin-induced respiratory depression and subsequent brain hypoxia. This alcohol-induced potentiation has been seen for different parameters in other opioids; in a study with human volunteers treated with alcohol and oxycodone, alcohol alone (0.5–1.0 g/L) induced minimal changes in respiration, but potentiated the respiratory depressive effects of oxycodone (20 mg) and caused a drop in oxygen saturation (68).

### 4.2 Ketamine–fentanyl

Ketamine is a short-acting general anesthetic with hallucinogenic, analgesic, and amnestic properties. Due to its hallucinogenic and tranquilizer properties, ketamine is often used by adolescents and young adults in party settings (69–71). Although safe when used in controlled doses by medical professionals, uncontrolled use of ketamine is dangerous, especially when mixed with other sedative drugs including alcohol, benzodiazepines, and opioids. In particular, synergistic antinociceptive interactions between opioids and ketamine have been documented in both preclinical and clinical studies (72–75), due to NMDA receptor antagonism and μ-opioid receptor agonism (76), potentially positing a greater health risk in cases of poly-drug use.

We examined the pattern of brain oxygenation responses induced by iv ketamine (3–27 mg/kg) and its co-administration with fentanyl (77). To explore the possible mechanisms underlying ketamine-induced brain oxygen responses, we also examined changes in temperature in the brain, temporal muscle, and skin, as well as changes in brain-muscle and skin-muscle differentials: indices that reflect changes in metabolic brain activity and peripheral vascular tone (78).

**Figure 7:**
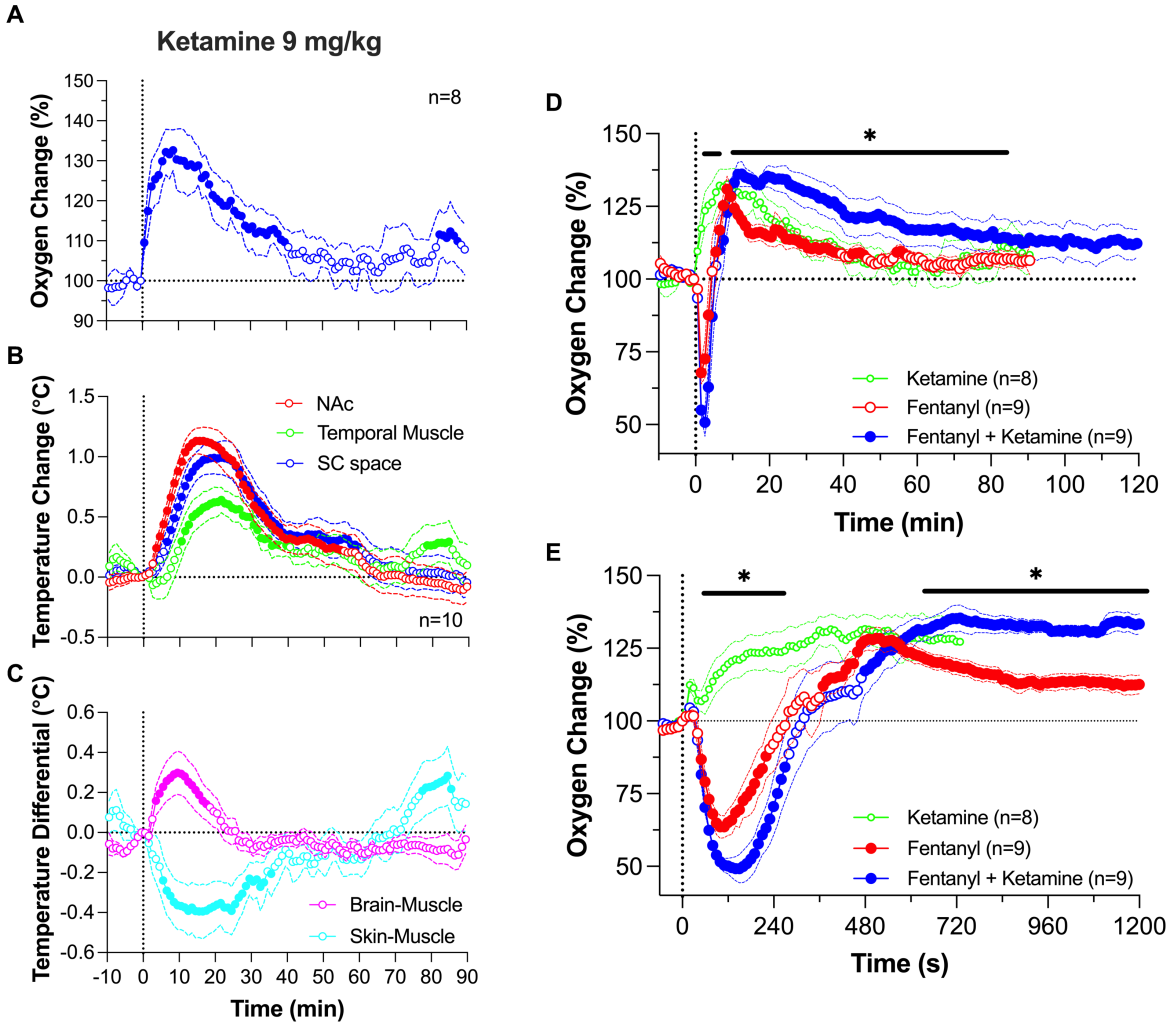
Changes in NAc oxygen levels induced by iv ketamine in freely moving rats. **(A)** shows NAc oxygen response induced by ketamine at a 9 mg/kg dose. **(B,C)** show changes in temperature in the NAc, temporal muscle and subcutaneous space as well as the changes in brain-muscle and skin-muscle differentials. **(D,E)** show changes in NAc oxygen induced by ketamine (9 mg/kg), fentanyl (0.02 mg/kg) and their mixture. Data were analyzed with slow **(D)** and rapid **(E)** time-course resolution. n is the number of averaged responses. Original data were published in Irwin et al. (77).

Ketamine (9 mg/kg; ~10% of a typical dose used for ketamine-xylazine anesthesia in rats) induced a rapid increase in NAc oxygen levels followed by a slower descent to the baseline (Figure 7A). Both the magnitude and duration of the oxygen increase were dose-dependent. Ketamine-induced increases in brain oxygenation were rapid and strong, preceding the slower increases in temperature at each recording location (Figure 7B). Ketamine also increased the brain-muscle differential, suggesting metabolic brain activation, and profoundly decreased the skin-muscle differential, suggesting skin vasoconstriction (Figure 7C). Despite quantitative differences, the pattern of ketamine-induced oxygen response was similar to that induced by tail-pinch (see Figure 1). Therefore, ketamine’s oxygen response mirrors that of an arousing stimulus by increasing metabolic brain activity, decreasing peripheral vascular tone, and inducing behavioral activation.

At a modest dose (9 mg/kg), the ketamine-fentanyl mixture induced a biphasic NAc oxygen response with a rapid and strong decrease followed by a more prolonged but weaker increase (Figure 7D). This pattern of this effect was similar to that induced by fentanyl alone, but the mixture potentiated both the initial hypoxic phase and the subsequent hyperoxic phase of fentanyl responses. A significant difference in oxygenation between the fentanyl response and the ketamine-fentanyl mixture response maintained from 11 to 85 min post-injection. In contrast to ketamine which increased locomotor activity, both fentanyl and the ketamine-fentanyl mixture induced severe hypoactivity, tail erection, and decreases in rate and depth of respiration, which was followed by a transient apnea during and immediately after injections. Muscle rigidity was present after fentanyl administration, but was often absent after injection of the ketamine-fentanyl mixture.

Between-group differences were additionally amplified following a rapid-time course analysis of the initial 20 min following the injection onset (Figure 7E). This analysis revealed that the oxygen drop is relatively short in duration in both groups, having identical onset latencies. However, in the ketamine-fentanyl group, the oxygen decrease was significantly larger than that with fentanyl alone from ~1 to ~5 min after the injection onset. As the oxygen response curve transitioned to the prolonged, hyperoxic phase, the mixture resulted in a significantly larger oxygen increase than that with fentanyl alone from ~10 min onward, reflecting the expected isolated effect of ketamine on brain oxygen levels.

This pattern of drug interaction is not explained by simple summation of drug effects. If the effects of drugs are summated, ketamine, which alone increases oxygen levels, should weaken the fentanyl-induced oxygen decrease. Instead, we observed that the hypoxic effect of fentanyl was exacerbated by the ketamine addition. However, the potentiated hyperoxic effect of drug mixture seems to exhibit elements of summation which may result from the greater hypoxic effect due to a compensatory mechanism following hypoxia. The peak of the ketamine-induced oxygen increase occurred at the same time as a peak of fentanyl-induced hyperoxia, and both curves then similarly decreased toward the pre-injection baseline. Due to the unusual pattern of oxygen changes induced by co-administration of ketamine and opioids, further study is needed to define the underlying mechanisms of this drug interaction.

### 4.3 Xylazine–heroin and fentanyl

The newest player in the US opioid epidemic is xylazine, which is a non-controlled substance traditionally used as a veterinary tranquilizer and component of general anesthesia in animals (79). Although xylazine is not approved for human use, a pattern of recreational use in the US has emerged in the past decade (80). Most alarmingly, xylazine has been found in a significant proportion of opioid-positive overdose deaths (25, 80), drawing attention to the need to establish the relationship between xylazine and opioids.

Xylazine is an agonist at the alpha-2 adrenergic receptors that induces sedation and muscle relaxation (81). At higher doses, it has been shown to significantly depress vital functions, causing strong hypotension, bradycardia, hypothermia, and respiratory depression (82). When taken with opioids that have similar physiological effects, the risk of overdose and death may increase. However, the mechanisms underlying the effects of xylazine and its interaction with opioid drugs remain relatively unknown.

**Figure 8:**
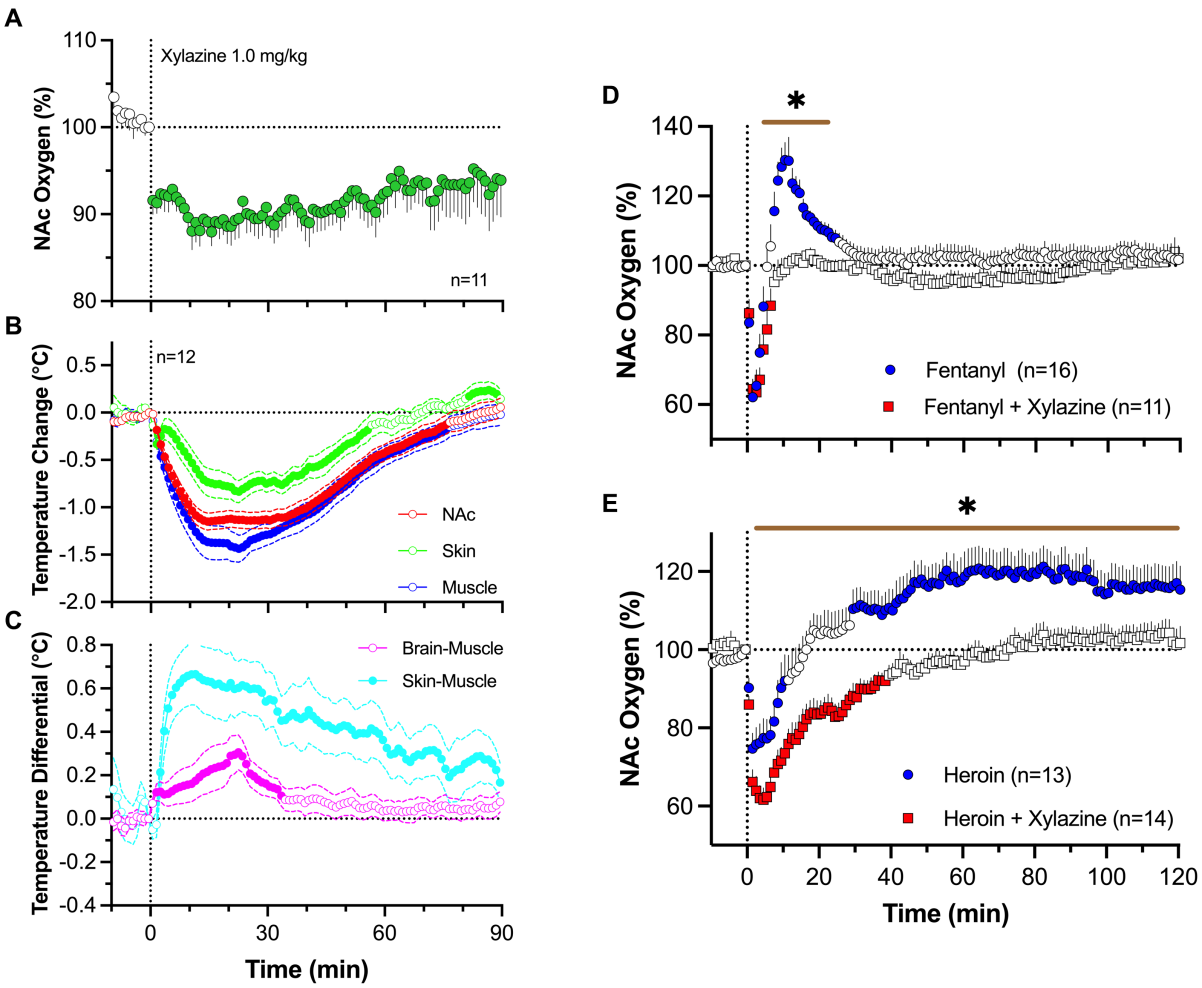
Changes in NAc oxygen levels induced by iv xylazine in freely moving rats. **(A)** shows NAc oxygen response induced by xylazine at 1 mg/kg dose. **(B,C)** show changes in temperature in the NAc, temporal; muscle and skin as well as changes in brain-muscle and skin-muscle differentials. **(D)** shows changes in NAc oxygen induced by iv fentanyl (0.02 mg/kg), and its mixture with xylazine (1 mg/kg). **(E)** shows changes in NAc oxygen induced by iv heroin and its mixture with xylazine. n is the number of averaged responses. Original data were published in Choi et al. (83).

To address this issue, we examined changes in brain oxygen induced by iv xylazine at different doses (0.3, 1.0 and 3.0 mg/kg) ranging from “safe” recreational use to possible overdose (83). As shown in Figure 8A, xylazine at a relatively low dose [1 mg/kg; 1:8 for rat general analgesic protocol and 1:30 for LD50 (84);] rapidly decreased NAc oxygen levels that followed with a slow ascent to the baseline. The largest drop in oxygen occurred within 10–30 s from the injection onset, i.e., within the duration of drug delivery.

By using three-point thermorecording, we found that xylazine decreases temperatures in the NAc, temporal muscle, and SC space (Figure 8B). Despite correlative changes, the decrease was strongest in temporal muscle, weaker in the NAc, and weakest in the SC space. Due to these differences, NAc-muscle and skin-muscle temperature differentials significantly increased, with weaker changes for the former and much stronger changes for the latter (Figure 8C). Therefore, xylazine induces strong peripheral vasodilation responsible for increases in heat loss and decreases in brain and body temperature. Although an increase in the brain-muscle differential induced by natural arousing stimuli indicates metabolic brain activation, this may not be applicable for xylazine, which induces muscle relaxation and atonia-related decreases in heat production (85). This mechanism may instead be responsible for the largest temperature decreases in the temporal muscle.

Xylazine (1 mg/kg) injected with fentanyl (20 μg/kg) induced a rapid brain oxygen decrease, which was similar to that induced by fentanyl alone (Figure 8D). However, it lacked the second hyperoxic phase of the oxygen response that was occurred with fentanyl alone. Due to disappearance of this second phase of the oxygen response, the total duration of oxygen decrease was much longer with the drug mixture than with the fentanyl alone. Both fentanyl alone and its mixture with xylazine induced similar behavioral effects, including severe hypoactivity, muscle rigidity in the limbs, and tail erection, as well as decreases in rate and depth of respiration.

Similar but more pronounced changes in the brain oxygen response were found with the mixture of heroin and xylazine. Heroin (0.6 mg/kg) administered alone induced a robust and prolonged decrease in NAc oxygen levels followed by a weaker, more prolonged oxygen increase (Figure 8E). The xylazine-heroin mixture also more strongly decreased brain oxygenation, and this decrease was more prolonged than with heroin alone. In contrast to the biphasic response for heroin alone, the second phase of oxygen response was absent after injection of heroin-xylazine mixture.

Hence, the danger of xylazine as an adulterant to opioid drugs appears to be related to prolongation of brain hypoxia due to blockade of the second, hyperoxic phase of the oxygen response induced by opioid drugs. While strong decreases of brain oxygen levels could be tolerated if they are transient, the harmful effects on brain cells are greatly enhanced when hypoxia is prolonged.

### 4.4 Factors affecting the therapeutic potential of naloxone

Naloxone, a potent opioid antagonist, is the primary agent used to alleviate respiratory depression induced by opioid drugs in intoxicated individuals (6, 86, 87). However, clinical data suggest that this therapeutic strategy is less effective with respect to fentanyl (22, 23, 88). Although insufficiency of dose may contribute to limited therapeutic effectiveness, the timing of naloxone treatment is the more critical variable, since fentanyl has rapid pharmacokinetics (89–91) and produces a powerful but transient effect (89, 91). In clinical settings, there is always a delay between the appearance of overdose symptoms and initiation of naloxone treatment. Thus, better understanding of the time-sensitive brain hypoxia induced by fentanyl and its relation to the timing of naloxone intervention appears to be of critical importance.

**Figure 9:**
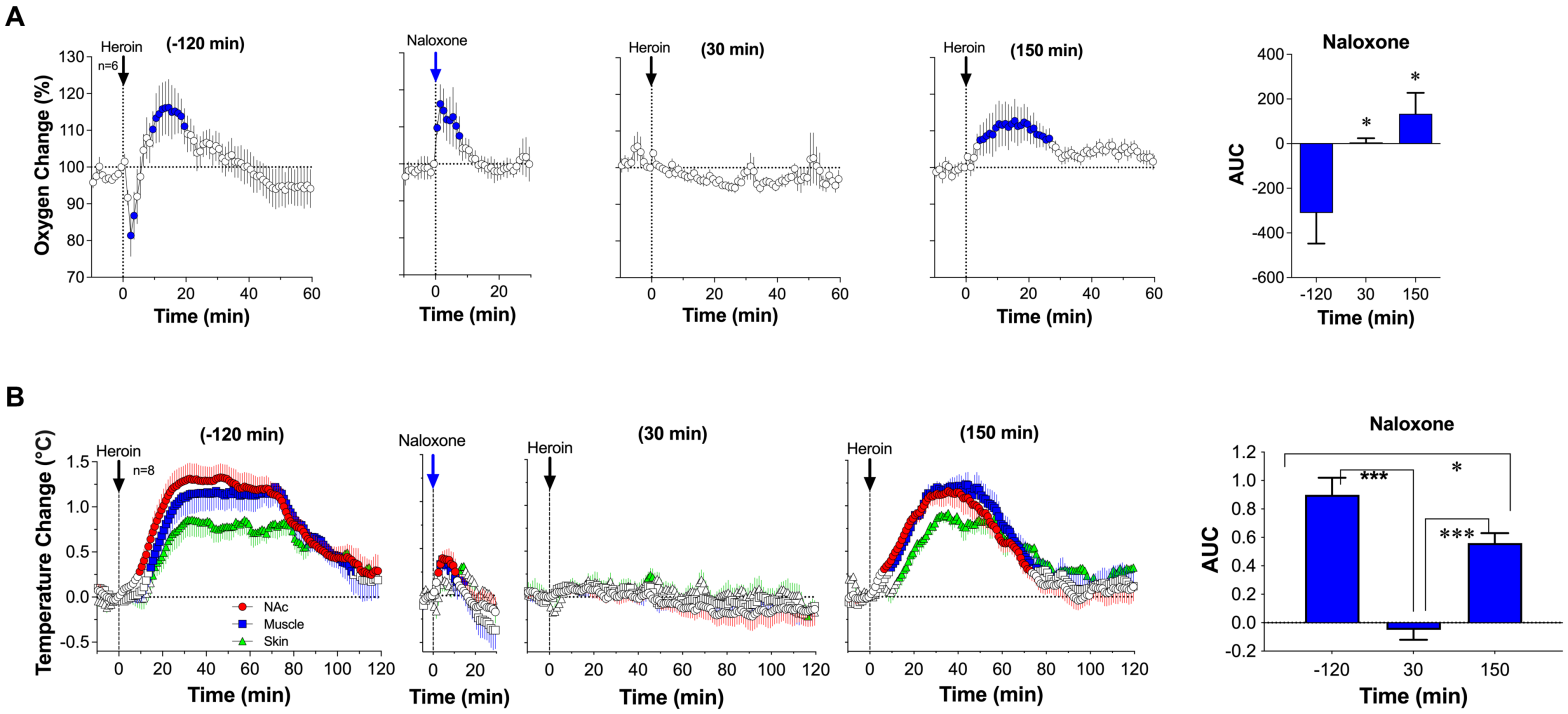
Changes in NAc oxygen levels **(A)** and temperatures **(B)** induced by iv heroin (100 μg/kg) before and after sc injection of naloxone (0.2 mg/kg). Original data were published in Perekopskiy et al. (92).

We first examined the effects of naloxone delivered subcutaneously at a relatively small, clinically relevant dose (0.2 mg/kg or 14 mg/70 kg) on brain oxygen responses induced by iv heroin (0.1 mg/kg) (92). At this dose, naloxone induces robust blockade of mu-opioid receptors (93). In our experiment, pre-treatment with naloxone fully blocked the biphasic effect of heroin (Figure 9A). Oxygen levels remain unaffected at 30 min after naloxone administration and the only modest increase was seen 150 min after naloxone injection. Naloxone at the same dose also fully blocked the temperature effects of heroin (Figure 9B). In contrast to hyperthermic effects of heroin, temperature did not change during the second heroin injected at 30 min after naloxone injection. The slightly weaker hyperthermic effects reappeared after the third injection made 150 min after naloxone. Naloxone injection itself induced a transient increase in both brain oxygen and temperatures; these effects can be attributed, in part, to the stress of subcutaneous drug injection.

**Figure 10:**
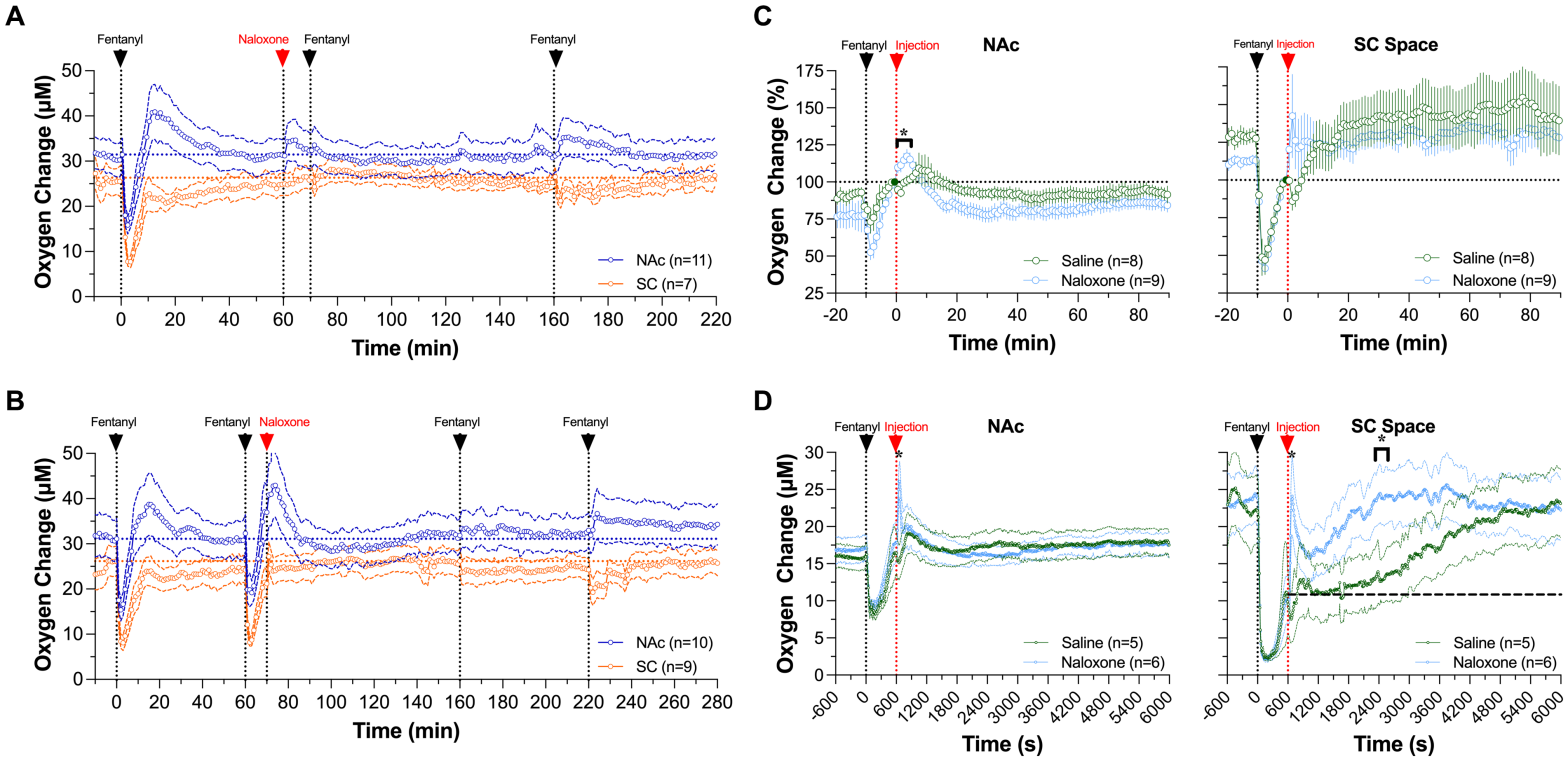
Mean changes in NAc **(A)** and SC oxygen **(B)** levels induced by fentanyl (20 μ/kg) before and after iv injection of naloxone (0.2 mg/kg). **(C,D)** show differences in the effects of naloxone and saline on NAc delivered 10 min after fentanyl injection (0.02 mg/kg and 0.06 mg/kg). Filled symbols show values significantly different from pre-injection baseline. Original data were published in Curay et al. (51).

Naloxone also fully blocked the oxygen response induced by iv fentanyl (20 μg/kg) (Figure 10A). Naloxone in this case was delivered iv at the same dose (0.2 mg/kg), and fentanyl was injected at 10 and 100 min after naloxone. In contrast to the fentanyl-induced biphasic oxygen response seen in the NAc, no change in baseline was observed for the fentanyl injection 10 min after naloxone injection and only a weak increase occurred after the fentanyl injection delivered 100 min after naloxone. Naloxone also fully blocked the oxygen response in the SC space. Naloxone administered alone seemed to increase brain oxygen levels slightly above the baseline for ~5 min after the injection. It is unlikely that these minor changes are induced by the drug; they could be explained by inescapable sensory influences associated with the procedure of iv injection.

We also examined the effect of naloxone on fentanyl-induced changes in oxygen levels in the NAc and SC space when administered after iv fentanyl injection (51). While it is known that naloxone should be delivered as early as possible, we picked a 10 min time interval between the onset of fentanyl delivery and start of naloxone injection to mimic a clinical response to overdose treatment. In this experiment, naloxone failed to induce an effect on the patterns of oxygen responses induced by fentanyl in both recording locations, since it was delivered after the hypoxic phase of the oxygen response had already ceased (Figure 10B). However, oxygen responses induced by the second and third fentanyl injections were fully blocked due to blockade of opioid receptors by naloxone.

To isolate the effect of naloxone injected 10 min post-fentanyl, we compared oxygen changes between oxygen responses after a naloxone or saline injection (Figure 10C). We found similar responses after both injections at both locations, with concentration curves superimposed over the entire 90 min after injections of both naloxone and saline. There were no significant between-drug differences, indicating that naloxone in our rat model has no effect on fentanyl-induced changes in oxygen levels when administered after cessation of the hypoxic phase. However, a minimal effect of naloxone, a stronger oxygen increase in both the NAc and SC space, was found within several minutes following its injection.

To test if there could be a dose-dependent difference in fentanyl-induced oxygen response, we examined the effects of naloxone and saline injections on oxygen responses induced by fentanyl at a larger dose (60 μg/kg). At this dose, naloxone caused a quicker return to baseline oxygenation than saline in both the NAc and SC space (Figure 10D). In the SC space, the naloxone-induced increase was rapid, followed by a second decrease, and then returned to the pre-fentanyl baseline. The total duration of hypoxia (~20 min) was much shorter than that after saline injection (~90 min). In the NAc, a significant difference between saline and naloxone appeared for a briefer time period after the injection. Naloxone administration caused NAc oxygen levels to rapidly increase above the pre-injection baseline within several minutes, while no such increase was seen after saline injection. Nevertheless, by the time naloxone and saline were injected at 10 min after fentanyl, NAc oxygen levels had already returned to the pre-injection baseline.

From this experiment, we concluded that the timing of naloxone delivery (or therapeutic window) is the most critical parameter in determining treatment efficacy. Although the hypoxic effects of fentanyl in the brain are strong and dose-dependent, they are relatively short at both lower and higher drug doses (~6 and 10 min for 20 and 60 μg/kg, respectively). Due to the short duration of hypoxia and subsequent post-hypoxic oxygen increase, delayed naloxone treatment is ineffective. Although the therapeutic window of naloxone should be larger in humans, time-sensitive delivery of naloxone remains crucial in treating individuals experiencing overdose.

The therapeutic potential of naloxone may also be diminished by poly-drug use, or contamination of opioid drugs by other neuroactive drugs. These adulterants may have their own hypoxic effects or block post-hypoxic vascular effects in the brain that exacerbate opioid-induced respiratory depression and potentiate brain hypoxia. In specific, we examined this issue by exploring the interaction of fentanyl and xylazine (94), an increasingly detected drug of abuse in the US illicit drug market.

**Figure 11:**
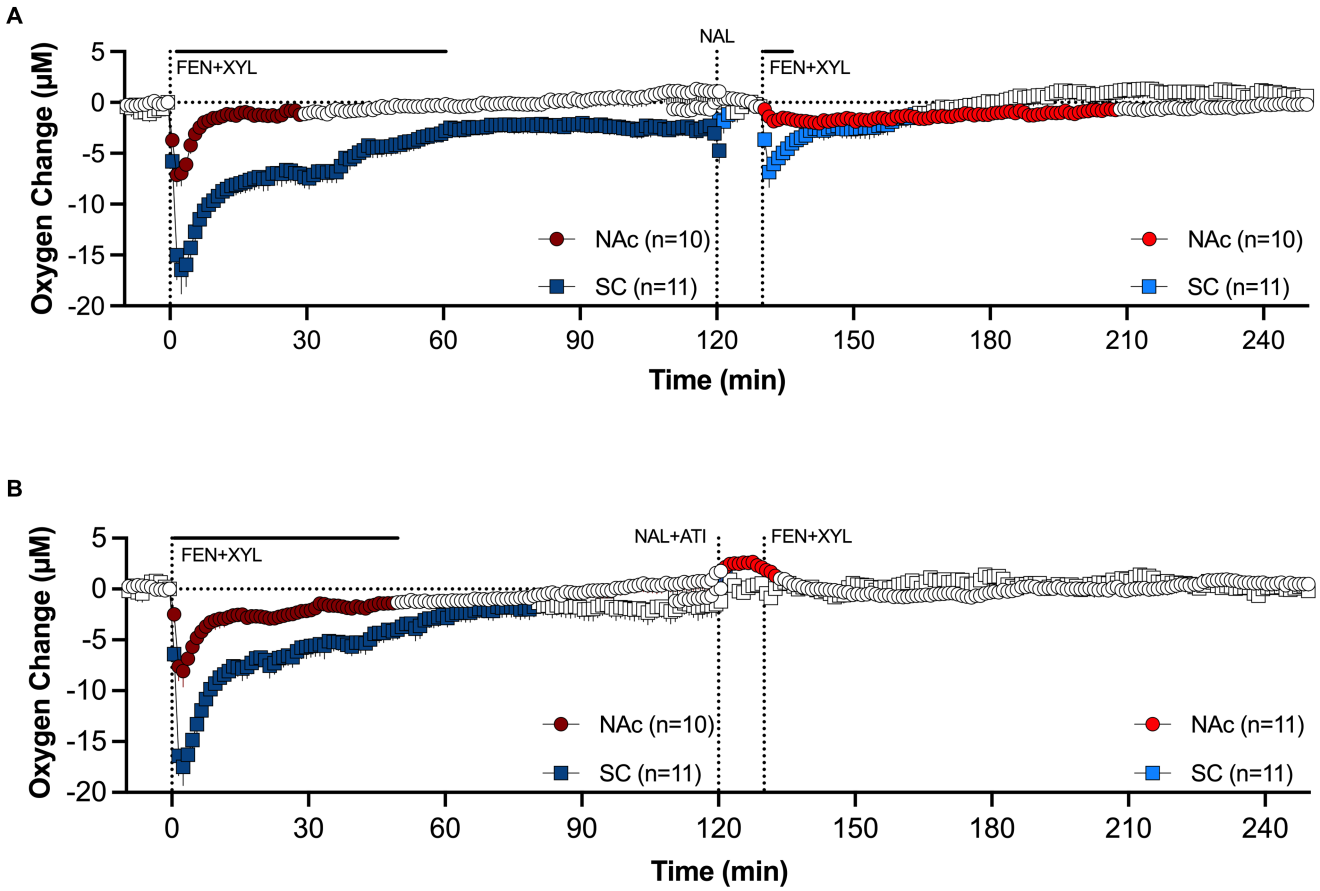
Mean changes in oxygen levels in the NAc and SC space induced by fentanyl+xylazine mixture before and after injections of naloxone **(A)** and naloxone-atipamezole mixture **(B)**. Antagonists were injected 10 min before agonists. Compiled from data published in Choi et al. (94).

In our experiment, naloxone, which effectively blocked the effects of fentanyl when injected pre-fentanyl (see Figure 10A), failed to attenuate hypoxia induced by a mixture of fentanyl and xylazine (Figure 11A). The attenuating effect was stronger in the brain and minimal in the SC space. In contrast to naloxone alone, a mixture of naloxone with atipamezole, a highly selective antagonist of alpha 2-adrenoceptors, fully blocked hypoxia induced by fentanyl-xylazine mixture (Figure 11B). These findings suggest that naloxone loses its therapeutic potential when fentanyl is mixed with xylazine, requiring additional use of antagonists that further reverse the effects of the adulterant.

**Figure 12:**
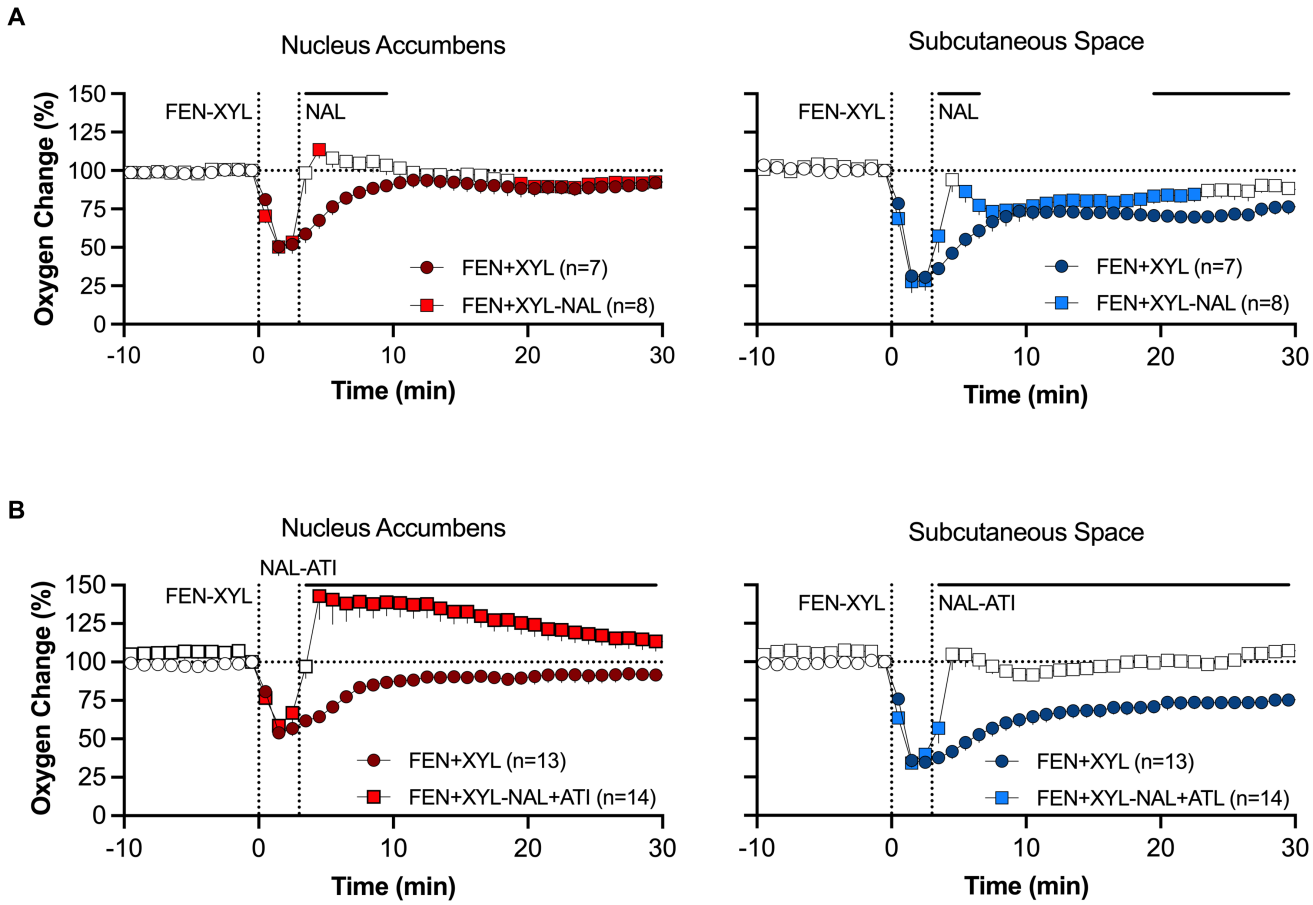
Mean changes in oxygen levels in the NAc and SC space induced by naloxone **(A)** and naloxone+atipamezole mixture **(B)** delivered 10 min after fentanyl+xylazine injection. Primary data were published in Choi et al. (94).

To model a scenario of overdose treatment, we also examined the effects of the naloxone alone and its mixture with atipamezole delivered at the peak of brain hypoxia induced by the fentanyl-xylazine mixture. The naloxone-atipamezole mixture rapidly increased oxygen levels in both locations, with a stronger effect in the brain (Figure 12A). Oxygen levels in this case strongly increased above baseline. Naloxone alone also induced a similar response, rapidly increasing oxygen levels to pre-injection baseline (Figure 12B). However, oxygen levels after sole naloxone treatment retained tonic oxygen decrease in both locations that was absent with the injection of the naloxone-atipamezole mixture.

Hence, although naloxone is the most effective drug for attenuating the effects of opioid drugs, its efficiency depends on three factors: drug dose, timing of delivery, and contamination with adulterants. Furthermore, though we believe our study is clinically relevant, our model is limited. Pharmacokinetic and pharmacodynamic effects of drugs differ between rodents and humans, naloxone is typically administered nasally which slows the drug’s onset of action in humans, and brain oxygenation may differ between rodents and humans due to metabolic or anatomical differences that affect vasculature. Hence, further study with alternative models will better translate for clinical interpretation.

## 5 Conclusion

Opioid abuse in the United States is a growing concern due to the high addictive potential of opioid drugs and their potent depressive effects on respiratory activity. As respiratory depression leads to brain hypoxia that may result in serious health complications and death, here, we have focused on and reviewed the mechanisms behind the brain oxygen dynamics following the exposure of freely moving rats to natural physiological stimuli, opioids (heroin and fentanyl), and xylazine-adulterated opioid mixtures, as well as during polysubstance use with alcohol and ketamine. In contrast to plethysmography that assesses drug-induced depression of respiratory activity or pulse oximetry that reveals blood oxygen decreases, electrochemical oxygen sensors allowed us to examine the patterns of physiological and drug-induced oxygen responses and reveal qualitative differences in oxygen dynamics in the brain and periphery. Specifically, both heroin and fentanyl at modest doses induced a biphasic pattern of brain oxygen response that displayed an initial rapid, strong but relatively transient decrease (hypoxia) followed by a subsequent weaker and more prolonged increase (hyperoxia), indicating the involvement of post-hypoxic compensatory vascular response. This biphasic pattern of brain oxygen response differs from the monophasic oxygen decreases we observed in the periphery and the more tonic decreases in respiratory activity obtained by plethysmography. We also showed that the hypoxic effects of heroin and fentanyl are potentiated or otherwise altered by the addition of alcohol, ketamine, and xylazine, solidifying a link between adulterated drug supply and heightened health complications. Finally, we considered the effectiveness of naloxone treatment in attenuating or blocking the hypoxic effects of opioid drugs, with a focus on the importance of timing of naloxone delivery and the presence of adulterants in opioid mixtures. By applying our findings, further study of drug interactions and opioid-related hypoxia in human subjects may aid in addressing health hazards and lethality from opioid misuse.

## Author contributions

EK: Conceptualization, Supervision, Writing – original draft, Writing – review & editing. SC: Conceptualization, Investigation, Writing – original draft, Writing – review & editing.
